# Metabolites and Lipids Associated with Fetal Swine Anatomy via Desorption Electrospray Ionization – Mass Spectrometry Imaging

**DOI:** 10.1038/s41598-019-43698-2

**Published:** 2019-05-10

**Authors:** Marisol León, Christina R. Ferreira, Livia S. Eberlin, Alan K. Jarmusch, Valentina Pirro, Ana Clara Bastos Rodrigues, Phelipe Oliveira Favaron, Maria Angelica Miglino, R. Graham Cooks

**Affiliations:** 1 Surgery Department, School of Veterinary Medicine and Animal Science, University of Sao Paulo, Sao Paulo, Brazil; 2 Department of Chemistry and Center for Analytical Instrumentation Development, Purdue University, West Lafayette, IN 47907 United States; 3 Department of Chemistry, The University of Texas at Austin, Austin, TX 78712 United States; 4 Collaborative Mass Spectrometry Innovation Center, Skaggs School of Pharmacy and Pharmaceutical Sciences, University of California San Diego, La Jolla, CA 92093 United States; 5 State University of Londrina, Londrina, Paraná 86051-990 Brazil

**Keywords:** Organogenesis, Anatomy

## Abstract

Chemical imaging by mass spectrometry (MS) has been largely used to study diseases in animals and humans, especially cancer; however, this technology has been minimally explored to study the complex chemical changes associated with fetal development. In this work, we report the histologically-compatible chemical imaging of small molecules by desorption electrospray ionization (DESI) - MS of a complete swine fetus at 50 days of gestation. Tissue morphology was unperturbed by morphologically-friendly DESI-MS analysis while allowing detection of a wide range of small molecules. We observed organ-dependent localization of lipids, *e.g*. a large diversity of phosphatidylserine lipids in brain compared to other organs, as well as metabolites such as N-acetyl-aspartic acid in the developing nervous system and N-acetyl-L-glutamine in the heart. Some lipids abundant in the lungs, such as PC(32:0) and PS(40:6), were  similar to surfactant composition reported previously. Sulfatides were highly concentrated in the fetus liver, while hexoses were barely detected at this organ but were abundant in lung and heart. The chemical information on small molecules recorded via DESI-MS imaging coupled with traditional anatomical evaluation is a powerful source of bioanalytical information which reveals the chemical changes associated with embryonic and fetal development that, when disturbed, causes congenital diseases such as spina bifida and cleft palate.

## Introduction

Molecular and anatomical features are intertwined. Yet they are often studied independently of each other, largely because of limitations in the available analytical tools. Animal models are essential to understanding disease including the development of new therapies in translational medicine. Since there are numerous anatomical, physiological and molecular similarities between pigs and humans and because pigs are frequently used as a source for organ transplantation^1^, studying pig ontogeny is an attractive choice for developmental biology research.

Lipids play an important role in cellular physiology and are implicated in developmental diseases such as type I diabetes^2,3^, Niemann–Pick diseases^4,5^ and Gaucher’s disease^5,6^. The physiological consequences of disrupting the genes responsible for lipid metabolism are often severe developmental problems including alterations in neural development, muscular dystrophy, bone deformity, cartilage developmental problems, altered respiratory system development, gonadal and reproductive dysfunctions, liver failure, and embryonic lethality^7–13^. The role of small metabolites and the processes which govern their formation, distribution, and roles are equally important and critical for proper anatomical and physiological development.

Mass spectrometry (MS) imaging is one method that provides spatial as well a chemical information – bridging the gap between anatomy and physiology. This combination allows acquisition of comprehensive morphological and chemical data and has the potential to contribute to lowering the number of animals needed in research. Desorption electrospray ionization – mass spectrometry (DESI-MS) is a matrix-free and simple chemical imaging method that can be used to map small molecules in unmodified tissue sections. In a DESI-MS imaging experiment, a spray of charged solvent droplets rasters across the surface of a sample (such as a tissue section placed on a glass slide) and molecules are dissolved in the solvent spot forming a microfilm. The DESI spray is accelerated by a nitrogen gas stream which drives the solvent droplets towards the sample. The subsequent droplets impact the liquid spot and release secondary microdroplets containing the desorbed molecules and direct them to the inlet of the mass spectrometer where solvent evaporates generating free gas-phase ions^14–16^. The application of DESI-MS to tissue analysis benefits from the use of histologycally-compatible solvent combinations, such as acetonitrile and dimethylformamide mixed at equal amounts. Such solvents maintain tissue integrity and cell morphology when used as the spray solvent in the DESI-MS analysis^17^. Morphologically-friendly DESI-MS imaging is used to profile metabolites and lipids present in tissue samples without disturbing the tissue morphology^17^. The preservation of morphology allows morphological information to be combined with data on the same tissue as is analyzed by DESI-MS^18,19^.

Lipid profiling and imaging, i.e. untargeted lipid detection and relative quantification, has been explored by DESI-MS and matrix-assisted laser desorption ionization (MALDI) MS in bovine, mouse, fish and swine animal species. However, these studies were restricted either to early embryonic stages (preimplantation) or to embryonic uterine implantation^16,20–29^. The use of the DESI-MS imaging for whole body analysis has been reported by Perez *et al*.^30^ to describe the location of toxic ionic liquids in specific areas of adult zebrafish. DESI-MS has allowed mapping of the location at 2D and 3D spatial domain for a wide range of lipid species such as free fatty acids (FFA), glycerophospholipids, glycerolipids and sphingolipids^22,31^.

In this research, we apply morphologically-friendly DESI-MS imaging combined with optical microscopy to characterize the location of FFA and some phosphatidylcholine (PC), phosphatidylserine (PS), sulfatide (ST), and phosphatidylinositol (PI) lipids in the whole body of two swine fetuses at an advanced stage of development (around day 50) when organogenesis can be observed. The location of selected molecules was studied in the 2D and 3D images. Since the lipid composition during mammalian organogenesis is largely unexplored, our findings were correlated with small molecule imaging reported for cells and organs. Lipids and metabolites concentrated in specific organs are pointed out since these may have key roles for the establishment of physiological function. As examples, palmitic acid, oleic acid, PC(36:1)/PE(40:4), and PE(40:3) were present in all organs. The nervous system showed high abundances of an array of specific PI and PS lipids while sulfatides were mostly detected in the gastrointestinal system. N-acetyl-L-glutamic acid was detected only in the heart, and N-acetyl-aspartate was specifically located in the brain tissue. To our knowledge, this is the first study describing the location of lipids and metabolites at an advanced stage of fetal development. DESI-MS imaging is highly applicable to understanding complex developmental processes and should be useful to study developmental diseases.

## Material and Methods

### Chemicals

When not otherwise stated, reagents were purchased from Sigma-Aldrich (St. Louis, MO). All reagents were HPLC grade (OmniSolv®).

### Samples

**Figure 1:**
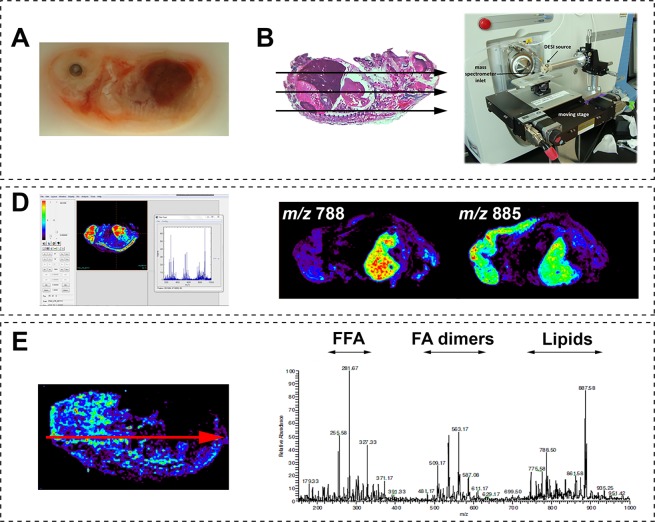
Workflow of DESI-MS imaging of a whole swine fetus. **(A)** Samples were sectioned using a cryotome and mounted onto glass slides. The whole swine fetus volume was sectioned using 300 µm of lateral resolution (Fig. S1). Glass slides were stored at −80 °C and before analysis were thawed and placed in a desiccator for ~15 min. **(B)** DESI-MS images were acquired in an ion trap mass spectrometer equipped with an in-house built moving stage. **(C)** Ion images were visualized using BioMAP 3.7.5.5 provided by Novartis Institutes for BioMedical Research (freeware). **(D)** Spectral data were averaged across an entire line of an ion image (red line) and the resulting DESI-MS mass spectrum was displayed, showing the mass-to-charge (*m/z*) range.

The experimental workflow is summarized in Fig. 1. For DESI-MS experiments, two frozen swine fetuses around day 50 of pregnancy were obtained from a commercial supplier (Animal Technologies, Inc.). No institutional committee is reported for this research since the biological samples used in the experiments were sold as frozen samples by Animal Technologies, Inc. No handling of live vertebrates occurred and therefore the experiments did not require approval from the Purdue Animal Care and Use Committee (PACUC). The samples were frozen and embedded in optimal cutting temperature (OCT) compound for slicing 15-μm thick sections using a cryotome (Shandon SME Cryotome cryostat GMI, Inc., Ramsey, MN, USA). Whole body sections were mounted onto plain glass slides (Erie Scientific, Portsmouth, NH). No fixative agents were used before or after slicing. After sectioning, the samples were stored at −80 °C until analysis, when they were dried in a desiccator for ∼15 min. All DESI-MS experiments were performed at Purdue University, IN.

### DESI-MS imaging and data analysis

Experiments were carried out in the negative ion mode by applying a −5 kV spray voltage to the needle of the syringe used to deliver the spray solvent^32^. Dimethylformamide-acetonitrile (1:1) (*v/v*) solvent was sprayed at the rate of 1.5 μL/min under 180 psi of nebulizing gas (N_2_) pressure. Mass spectra were acquired from *m/z* 150 to 1,000 from different sections representing the whole swine fetus body. The DESI spray was at an incident angle of 51° to the surface plane and *circa* 2 mm distant from the instrument inlet. The DESI spray emitter consisted of an inner capillary with an inner diameter (ID) of 50 μm and outer diameter (OD) of 150 μm, and an outer capillary with an ID of 250 μm and an OD of 350 μm. The mass spectrometer used was an LTQ linear ion trap controlled by Xcalibur 2.0 software (Thermo Fisher Scientific, San Jose, CA, USA). Tissues were scanned using a lab-built 2D moving stage with horizontal rows separated by a vertical step of 300 μm. An in-house program allowed the conversion of the XCalibur 2.0 mass spectra files (.raw) into a format compatible with Biomap (freeware, http://www.maldi-msi.org), in which spatially resolved images were assembled and displayed in the interpolated mode. Ion images were independently normalized to 100 and displayed using a color-coded scale. The lipid species were putatively identified by MS/MS and by high mass resolution obtained by manually directing the DESI spray onto specific organs using an Orbitrap mass spectrometer (Exactive, Thermo Scientific, San Jose, CA, USA). After DESI-MS imaging, the whole-body tissue sections were stained with H&E and morphological images were manually overlaid with the DESI-MS ion images. Biomap (Novartis, Basel, Switzerland) was used for the visualization of 2D ion images, and a MATLAB-based data processing routine was used for 3D mass spectrometry imaging^31^. Due to the wealth of molecular location information acquired by DESI-MS imaging, not all data could be presented in this manuscript. The authors will gladly share all data upon request.

## Results

Macroscopic evaluation was used to determine gestational age. Swine fetuses used for this study had 37 mm of crown-rump indicating a gestational age of approximately 50 days^32^. Organogenesis stage was determined by morphology as described elsewhere^33^ and supported by the complete formation of the eyes, heart, brain, lungs, liver, stomach, and kidneys. Further evaluation revealed cephalic regions, eyes with pigmented retinas, upper and lower eyelids, external ears, and limbs with keratinized and separated digits. Additional details on the morphological evaluation are shown in Figs S2 and S3.

**Figure 2:**
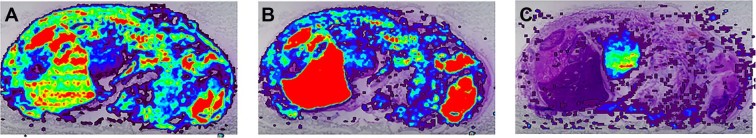
Overlay of 2D DESI-MS image and post-hoc H&E stain. (**A**) *m/z* 215; (**B**) *m/z* 885; and (**C**) *m/z* 788.

The DESI-MS imaging experiment was used to visualize the 2D and 3D molecular anatomy of the fetus associated with developmental processes (see discussion). The lateral resolution used was 300 µm, which allowed most of the organs to be evident while maintaining a reasonable data acquisition of 3 h for the largest whole-body 2D image. The 3D volumetric distribution included 2D images of 45 tissue sections (longitudinal sections spaced by an average of 160 µm) of one of the swine fetuses. The imaging experiment is not destructive, allowing for posthoc histopathology and co-registration of chemical and morphological information (Fig. 2).

**Figure 3:**
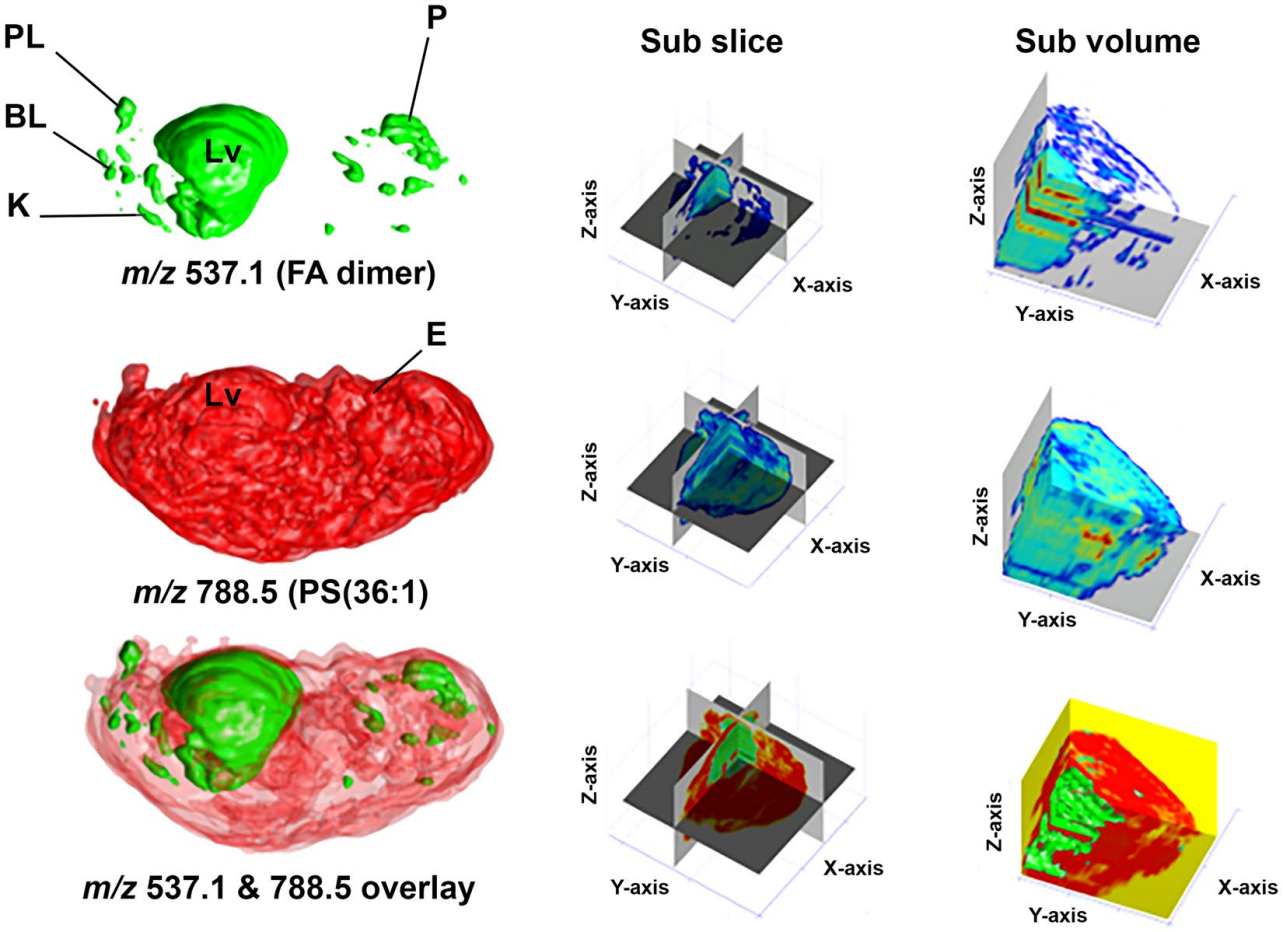
3D mass spectrometry images of C18:1 and C16:0 FA dimer (*m/z* 537), and of PS(36:1) *m/z* 788 generated by aligning 2D ion images of 45 tissue sections to illustrate the distribution of these molecules in the whole fetus. These images were obtained using the software package described previously^31^. Legend: (Lv) Liver, (P) Prosencephalon, (PL) Pelvic limb, (BL) Blowel loops, (K) Kidney, (E) Eye.

Figure 3 displays as example the location in 3D of the FA dimer C16:0 and C18:1, *m/z* 537, overlaid with the location of PS(36:1), *m/z* 788. Higher ion abundances for the FA dimer were found in the liver and the proencephalic mass (diencephalon and telencephalon) of the nervous system as well as in the intestines, while the PS(36:1) is distributed volumetrically throughout the entire body except the intestines. The creation of 3D images from the 2D DESI images illustrates the power of MS imaging for morphological and developmental studies, providing complementary chemical whole-body mapping. The 3D visualization provides information on how the compound distribution changes over the body volume. Examples of 3D reconstruction from 2D images by DESI-MS data are shown by Eberlin *et al*. (2010) and Xiong *et al*. (2012) for mouse brain^31,34^. Dueñas *et al*. (2017) reported 3D zebrafish imaging by MALDI-MS^28^.

**Figure 4:**
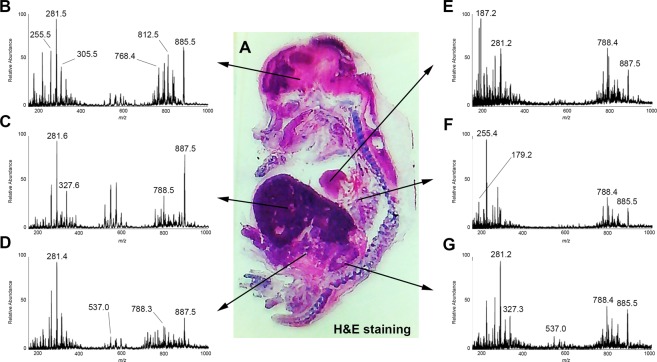
Representative mass spectra of some of the organs (indicated in the same H&E stained tissue section used for the DESI-MS imaging) and selected ion images. (**A**) H&E; (**B**) brain; (**C**) liver; (**D**) intestine; (**E**) heart; (**F**) lungs; (**G**) kidney.

**Figure 5:**
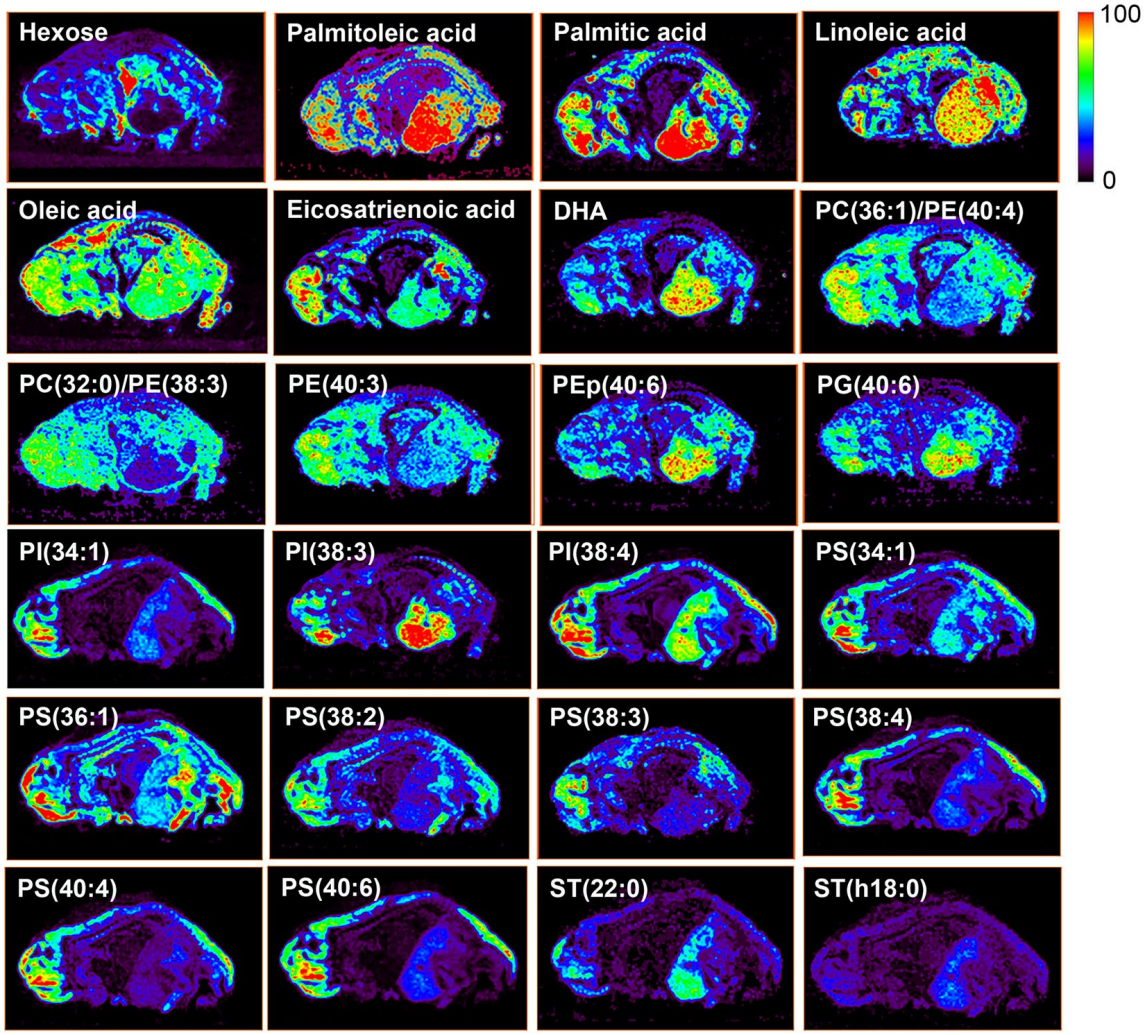
Ion images of selected metabolites and lipids distributed in the whole swine fetus body. Abundance in each ion image is independently scaled to 100 using the jet color-scale. Attributions are listed in Table 1. All ions are deprotonated versions of the molecules, viz. [M − H]^−^, except for PC lipids which were detected as chlorine adducts [M + Cl]^−^ in negative ion mode^63,64^.

**Figure 6:**
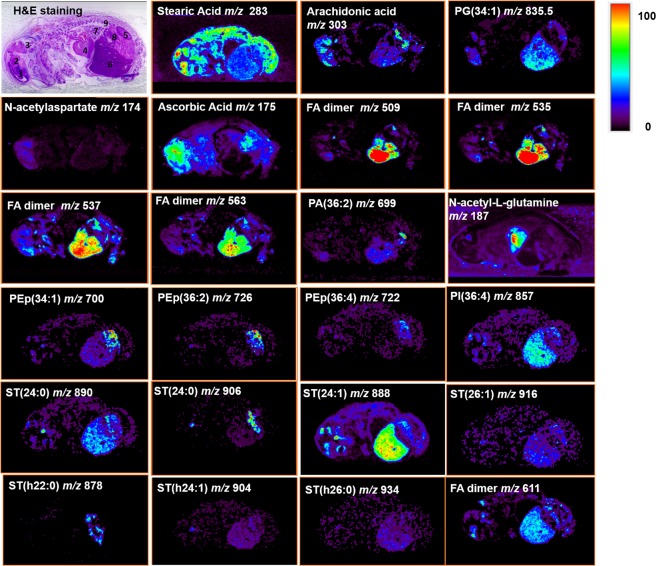
The first figure is an H&E micrograph with numbers indicating the organ location: 1. Proencephalon, 2. Mecencephalon, 3. Rhombencephalon, 4. Heart, 5. Kidney, 6. Liver 7. Lungs, 8. Digestive system. The selected ion images show the distribution of specific free fatty acids, metabolites and lipids in the fetus whole body. Abundance in each ion image is independently scaled to 100 using the jet color-scale. Attributions are listed in Table 1. All ions are deprotonated versions of the molecules, viz. [M − H]^−^, except for PC lipids which were detected as chlorine adducts [M + Cl]^−^.

Table 1 lists a set of lipids and metabolites that were detected in the nervous system (brain and spinal cord), cardiopulmonary system (heart and lungs), gastrointestinal and urinary systems (liver, intestines, and kidneys). The lipids were detected in negative ion mode, mostly as deprotonated ions [M − H]^−^ with the exception of phosphatidylcholines (PCs) and ceramides that were detected as chlorinated adducts [M + Cl]^−^ ^35^. Other lipids classed included phosphatidylserines (PS), sulfatides (ST), phosphatidylinositols (PI), sphingomyelin (SM), and FFA, all of which have structural and signaling roles in cells. Illustrative mass spectra for each organ are shown in Fig. 4. Putative metabolite and lipid annotations were based on DESI-MS literature, tandem MS and high-resolution MS data (Table 1, Fig. S4). Figures 5 and 6 depict ion images of selected lipids and their differential distribution in 2D throughout the swine fetus. Ion intensities in the images are color-coded and scaled to 100. Intensities are scaled independently in each ion image to optimize the contrast and facilitate comparison with morphological distribution.

**Table 1:** DESI high resolution mass spectral data and tentative identifications.

Identification	Ion	Chemical formula (Ion)	Theoretical mass	Measured mass	Mass Error (ppm)
N-acetyl-aspartate	[M − H]^−^	C_6_H_8_NO_5_	174.03981	174.03981	0
Ascorbic acid	[M − H]^−^	C_6_H_7_O_6_	175.02371	175.02385	0.8
C6 sugar	[M − H]^−^	C_6_H_11_O_6_	179.05501	179.05515	0.8
N-acetyl-glutamine	[M − H]^−^	C_7_H_11_N_2_O_4_	187.07133	187.07122	0.6
Palmitoleic acid	[M − H]^−^	C_16_H_29_O_2_	253.21621	253.21644	0.9
Palmitic acid	[M − H]^−^	C_16_H_31_O_2_	255.23186	255.23208	0.9
Linoleic acid	[M − H]^−^	C_18_H_31_O_2_	279.23186	279.23201	0.5
Oleic acid	[M − H]^−^	C_18_H_33_O_2_	281.24751	281.24766	0.5
Stearic acid	[M − H]^−^	C_18_H_35_O_2_	283.26316	283.26332	0.6
EicosaPentaenoic acid	[M − H]^−^	C_20_H_29_O_2_	301.21621	301.21630	0.3
Arachidonic acid	[M − H]^−^	C_20_H_31_O_2_	303.23186	303.23195	0.3
Eicosatrienoic acid	[M − H]^−^	C_20_H_33_O_2_	305.24751	305.24758	0.2
Eicosadienoic acid	[M − H]^−^	C_20_H_35_O_2_	307.26327	307.26316	−0.4
DHA (ω-3)	[M − H]^−^	C_22_H_31_O_2_	327.23186	327.23187	0.03
DPA (ω-3)	[M − H]^−^	C_22_H_33_O_2_	329.24751	329.24752	0.03
Eranthic acid (ω-6)	[M − H]^−^	C_22_H_37_O_2_	333.27881	333.27883	0.1
FA dimer	[M − H]^−^	—	—	509.1*	—
FA dimer	[M − H]^−^	—	—	535.2*	—
FA dimer	[M − H]^−^	—	—	537.2*	—
FA dimer	[M − H]^−^	—	—	563.2*	—
FA dimer	[M − H]^−^	—	—	611.3*	—
FA dimer	[M − H]^−^	—	—	699.3*	—
Cer(d34:1)	[M + Cl]^−^	C_34_H_67_NO_3_Cl	572.48040	572.47970	−1.2
Cer(d36:1)	[M + Cl]^−^	C_36_H_71_NO_3_Cl	600.51170	600.51102	−1.1
Cer(d42:2)	[M + Cl]^−^	C_42_H_81_NO_3_Cl	682.58995	682.58907	−1.3
PEP(34:1)	[M − H]^−^	C_39_H_75_NO_7_P	700.52752	700.52698	−0.8
PEP(36:4)	[M − H]^−^	C_41_H_73_NO_7_P	722.51192	722.51191	−0.01
PEP(36:2)	[M − H]^−^	C_41_H_77_NO_7_P	726.54322	726.54281	−0.6
PE(36:2)	[M − H]^−^	C_41_H_77_NO_8_P	742.53813	742.53745	−0.9
PG(34:1)	[M − H]^−^	C_40_H_76_O_10_P	747.51706	747.51626	−1.1
PeP(38:4)	[M − H]^−^	C_43_H_77_NO_7_P	750.54322	750.54249	−1.0
PeP(38:3)	[M − H]^−^	C_43_H_79_NO_7_P	752.55887	752.55796	−1.2
PS(34:1)	[M − H]^−^	C_40_H_75_NO_10_P	760.51231	760.51130	−1.3
PE(38:4)	[M − H]^−^	C_43_H_77_NO_8_P	766.53813	766.53727	−1.1
PE(38:3)	[M − H]^−^	C_43_H_79_NO_8_P	768.55379	768.55301	−1.0
PG(36:2)	[M − H]^−^	C_42_H_78_O_10_P	773.53271	773.53191	−1.0
PG(36:1)	[M − H]^−^	C_42_H_80_O_10_P	775.54836	775.54733	−1.3
PS(36:2)	[M − H]^−^	C_42_H_77_NO_10_P	786.52796	786.52708	−1.1
PS(36:1)	[M − H]^−^	C_42_H_79_NO_10_P	788.54361	788.54267	−1.2
PC(34:0)	[M + Cl]^−^	C_42_H_82_NO_8_PCl	794.54611	794.54508	−1.3
PS(38:4)	[M − H]^−^	C_44_H_77_NO_10_P	810.52796	810.52690	−1.3
PS(38:3)	[M − H]^−^	C_44_H_79_NO_10_P	812.54361	812.54272	−1.1
PS(38:2)	[M − H]^−^	C_44_H_81_NO_10_P	814.55926	814.55942	0.2
PG(40:6)	[M − H]^−^	C_46_H_78_O_10_P	821.53271	821.53160	−1.4
PS(40:6)	[M − H]^−^	C_46_H_77_NO_10_P	834.52796	834.52698	−1.2
PS(40:4)	[M − H]^−^	C_46_H_81_NO_10_P	838.55926	838.55841	−1.0
PS(40:3)	[M − H]^−^	C_46_H_83_NO_10_P	840.57491	840.57402	−1.1
PI(36:4)	[M − H]^−^	C_45_H_78_O_13_P	857.51746	857.51626	−1.4
PI(36:3)	[M − H]^−^	C_45_H_80_O_13_P	859.53186	859.53311	1.5
PI(36:2)	[M − H]^−^	C_45_H_82_O_13_P	861.54876	861.54809	−0.8
PI(36:1)	[M − H]^−^	C_45_H_84_O_13_P	863.56358	863.56441	1.0
ST(h22:0)	[M − H]^−^	C_46_H_88_NO_12_S	878.60217	878.59859	−4.1
PI(38:5)	[M − H]^−^	C_47_H_80_O_13_P	883.53311	883.53185	−1.4
PI(38:4)	[M − H]^−^	C_47_H_82_O_13_P	885.54876	885.54757	−1.3
PI(38:3)	[M − H]^−^	C_47_H_84_O_13_P	887.56441	887.56371	−0.8
(3′-sulfo)Galβ-Cer(d18:1/24:1(2OH))	[M − H]^−^	C_48_H_90_NO_12_S	904.61782	904.6*	—
(3′-sulfo)Galβ-Cer(d18:1/24:0(2OH))	[M − H]^−^	C_48_H_92_NO_12_S	906.63297	906.63347	0.6

*Measured in low resolution using a linear ion trap mass spectrometer.

## Discussion

### Lipid and metabolite composition of the swine fetal nervous system

The nervous system, particularly the brain, has been extensively studied using mass spectrometry imaging (DESI and MALDI). The lipid composition of the brain presents some difference between species^36,37^, but it is nominally consistent within each species. The nervous system displayed a great variety of metabolites, illustrated in Figs 5 and 6. Among those that stand out arachidonic acid (C20:4, Fig. S4), stearic acid (C18:0), PG(34:1; Fig. S4), and PS(36:1) were also identified by MALDI-MS during mouse embryo uterine implantation^20^.

Other metabolites identified in the brain such as palmitoleic acid (C16:1), palmitic acid (C16:0), oleic acid (C18:1), and PI(38:4) (Figs 5 and 6), besides the arachidonic acid (C20:4) they have also been previously identified by l DESI-MS in the brains of adult rats^38^. We also detected fatty acids such as linoleic acid (C18:2) and eicosatrienoic acid (C20:3), which have been related to the differentiation and proliferation of neuronal cells, and with the synaptic development and peripheral neuropathies in humans^39^. The swine fetus showed differential distribution of PI lipids, such as PI(34:1), PI(38:3) and PI(38:4) through the different organs, but overall these lipids were more abundant at the nervous system and liver (Fig. 5). Burnum *et al*.^20^ correlated the location of PI lipids during mouse embryonic implantation with the differentiation and proliferation of cells in the mouse embryo (rearrangement of the cytoskeleton, trafficking of intracellular vesicles). Girod *et al*.^40^ detected differential distribution of PI(38:4) and PS(36:1) by DESI-MS in the areas of brain white and gray matter and in the spinal cord of the adult mice.

N-Acetyl-aspartate (NAA) of *m/z* 174 was most abundant in the nervous system compared to the other organs (Figs 6; S4). NAA is the second most concentrated molecule in the brain after the amino acid glutamate^26^. This metabolite has been detected in the human and murine brain tissue and can be used as a biomarker of healthy brain tissue when imaging brain tumors^26,41^. Roles for NAA include neuronal osmoregulation and axon-glial signaling, brain nitrogen balance^42^. Two human inborn errors are related to NAA metabolism: Canavan disease in which there is a buildup of NAA and associated spongiform leukodystrophy, caused by a lack of aspartoacylase activity. The  later is a human condition  where lack of NAA where the enzyme that synthesizes NAA is absent^43^. Differences in lipid abundances were identified for the brain and spinal cord. The brain showed higher abundances than the spinal cord for ascorbic acid (Fig. 6), DHA (C22:6), PC(36:1)/PE(40:4), PC(32:0)/PE(38:3), PE(40:3), PEp(40:6), PG(40:6), PI(38:4), PS(40:4) and PS(40:6) (Fig. 5). DHA has been reported as the predominant fatty acid in the central nervous system and the retina in humans. It is also essential for the optimal functional maturation of retina and visual cortex, as well as in neural development^44^. DHA level decrease has been linked in aging with diseases such as dementia and Alzheimer’s disease^39^. Also FA dimers of *m/z* 509, *m/z* 535, *m/z* 537, *m/z* 563 and PA(36:2) of *m/z* 699 (Fig. 6) were noticeably more present in the brain than in the spinal cord. FA dimers are formed during the desorption/ionization process and are not endogenous species in the tissue. Their abundance in the mass spectrum is proportional to the quantity of free FA in the tissue^45,45,46^. The role of PIs in the nervous system lies in the formation of the neurite and the neural circuit^47^ and the development of dendrites and the neuronal synapses^48^.

Overall, the nervous system showed high abundances for six out of seven PS lipids observed, which may be related to neuronal apoptosis, a key mechanism in brain organogenesis^12,13^. Specifically PS(38:3) and PS(40:4) have been associated with human brain tumors by Eberlin *et al*.^49^ using DESI-MS and this is an interesting finding since glioblastoma tumorigenesis has been related to cellular dedifferentiation at embryological states^50^.

### Lipid and metabolite composition of the swine cardiopulmonary system

Hexoses were more evident in the heart than in other organs and barely detected in liver (Fig. 5). N-Acetyl-glutamine was strikingly abundant in the heart compared to any other organ (Fig. 6). The concentration of N-acetyl-glutamine in cardiac tissue may be related to the enzymatic activity of acetyl kinase in this organ. The absence of this enzyme causes atrophy and cardiac muscle weakness^51^. To our knowledge this is the first time that N-acetyl-glutamine has been reported to be concentrated in the developing heart tissue compared to other parts of the body.

Overall, the lipids of highest abundance for the lungs were PS(36:1) and PC(32:0)/PE(38:3) (Fig. 5). Some of the lipids present at the lungs could relate to the pulmonary surfactant beginning to be synthesized at this time of fetal development. Specifically PS is important for lung maturation and to prevent the collapse of alveoli in expiration^52,53^. According to Sozo *et al*.^54^, pulmonary surfactant is composed mostly of phospholipids such as PC(32:0), PC(36:1), PI(34:1), PI(38:3), PS(36:1) and PS(40:6). So except for PI(34:1) and PS(40:6), all these lipids were detected in the swine fetal lungs. Interestingly, swine and human surfactants show similar compositions and this fact has allowed the use of swine surfactant for the treatment of hyaline membrane disease in preterm newborns^55–57^.

### Lipid and metabolite composition of the swine fetus gastrointestinal and urinary system

The liver showed the highest abundances in the fetal body of the FA dimers of *m/z* 509, *m/z* 535, *m/z* 537 and *m/z* 611 (Fig. 6). PI(34:1), PI(38:4) and PA(36:2) were detected in the pig fetuses liver and also were reported in pig oocytes by DESI-MS^23,28^. In addition, PI(38:4) and PA(36:2) are considered biomarkers of connective tissue and hepatic parenchyma respectively, being identified in dogs and human liver by MALDI-MS imaging^58^.

The intestines were unique for PEp(36:2), PEp(36:4) and ST(h22:0) (Fig. 6). PI(36:4) was also detected in the intestines but absent in the renal system. Inglese *et al*.^59^ identified PG(40:6) of *m/z* 821 and PI(38:4) of *m/z* 885 (Fig. S4) in human colorectal adenocarcinoma biopsies by 3D-DESI-MS.

Ascorbic acid, palmitic (C16:0), palmitoleic (C16:1), stearic (C18:0), oleic (C18:1), linoleic (C18:2), stearic (C18:1), arachidonic (C20:3), and eicosatrienoic (C20:4) acids (Figs 5 and 6) were detected in the kidneys at high ion abundances; and the glycerophospholipids such as PG(40:6), PI(34:1), PI(38:3), PI(38:4), PS(34:1), and PS(36:1) (Fig. 5) were also detected in the kidneys^22^. Dill *et al*.(2010) mapped PI(38:4) and PS(36:1) in human renal cell carcinomas^60^. Pirro *et al*. (2012) reported the presence of PI(38:4) and PS(36:1) as markers of cancer in human bladder, kidney, germ cells and prostate cancer. We were not able to observe fully developed adrenal glands in porcine fetuses, but the lipids we observed were also identified with high abundances in the adult pig adrenal glands using DESI-MS^61^. Other lipids such as PI(34:1), PG(34:1) have been associated with human cell carcinomas using DESI-MS^62^.

## Conclusion

Small molecules detected by DESI-MS in tissue sections from whole swine fetuses showed organ-specific distributions, such as N-acetyl-glutamine in the heart, ST(ht22:0) in the intestines, PA(36:2) in the liver, and a number of PS lipids in the nervous system. The chemical information provided by DESI-MS imaging reflects physiology, adds complementary information to anatomical studies and indicates key lipids related to physiological organogenesis. We envisage that this approach can be used to understand inborn developmental errors, especially as related to rare congenital conditions, environmental and epigenetic factors, and due to the application of specific biotechnologies, such as nuclear transfer.

## Supplementary information

Supplementary Information

## Data Availability

The datasets generated during and/or analyzed during the current study are available from the corresponding author upon request.
