# Supplementary material for: Metabolites and Lipids Associated with Fetal Swine Anatomy via Desorption Electrospray Ionization – Mass Spectrometry Imaging
Source: Sci Rep. 2019 May 10;9:7247. doi: 10.1038/s41598-019-43698-2 (PMC6510765; doi:10.1038/s41598-019-43698-2)
Supplement: Supplementary file 1 — Supplementary Information [file 41598_2019_43698_MOESM1_ESM.docx]

**Supplementary Information**

**Metabolites and Lipids Associated with Fetal Swine Anatomy via Desorption Electrospray Ionization – Mass Spectrometry Imaging**

Marisol León^1^, Christina R. Ferreira^2^, Livia S. Eberlin^3^, Alan K. Jarmusch^4^, Valentina Pirro^2^, Ana Clara Bastos Rodrigues^1^, Phelipe Oliveira Favaron^5^, Maria Angelica Miglino^1^, R. Graham Cooks^2,*^.

^1^Surgery Department, School of Veterinary Medicine and Animal Science, University of Sao Paulo, Sao Paulo, Brazil.

^2^Department of Chemistry and Center for Analytical Instrumentation Development, Purdue University, West Lafayette, IN 47907, United States.

^3^Department of Chemistry, The University of Texas at Austin, Austin, TX 78712, United States.

^4^Collaborative Mass Spectrometry Innovation Center, Skaggs School of Pharmacy and Pharmaceutical Sciences, University of California San Diego, La Jolla, CA 92093, United States.

^5^State University of Londrina, Londrina, Paraná 86051-990, Brazil.

***Corresponding Author**

Prof. R. Graham Cooks

560 Oval Drive

West Lafayette, IN 47906

cooks@purdue.edu

| 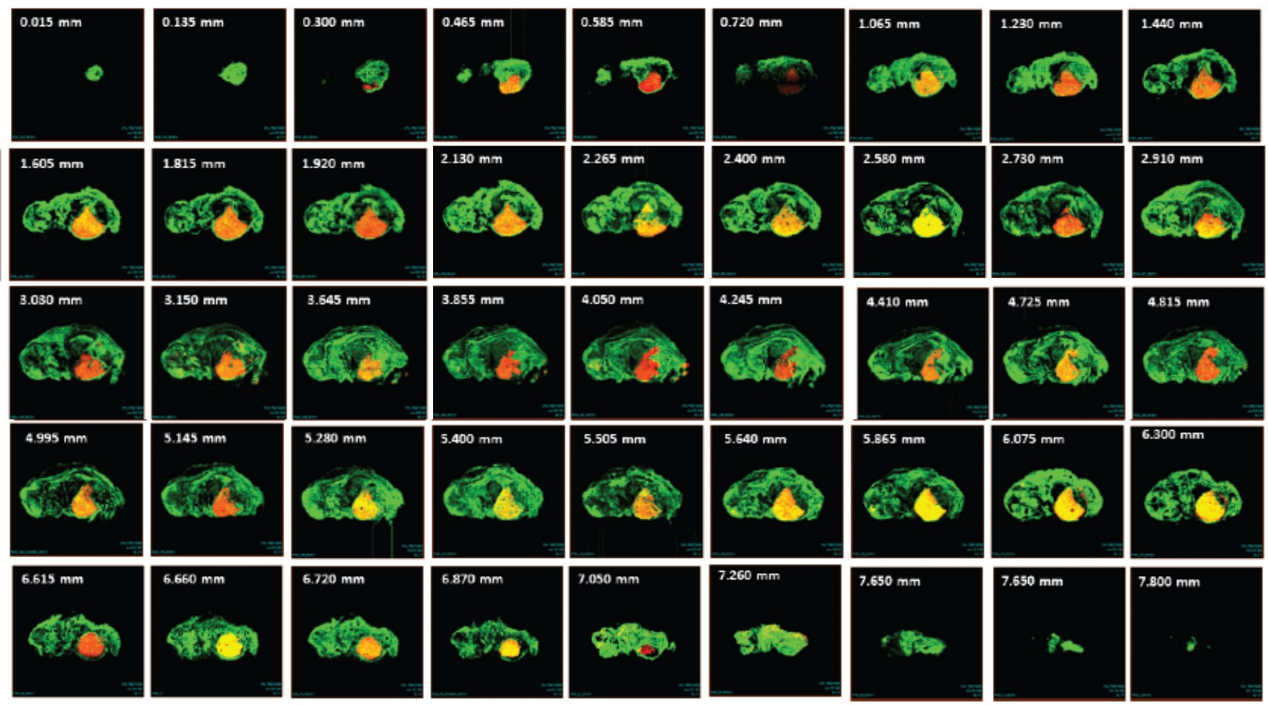 |
| --- |
| **Figure 1S**. Forty-five 2D overlaid ion images used to render a 3D model of the pig fetus. |

**
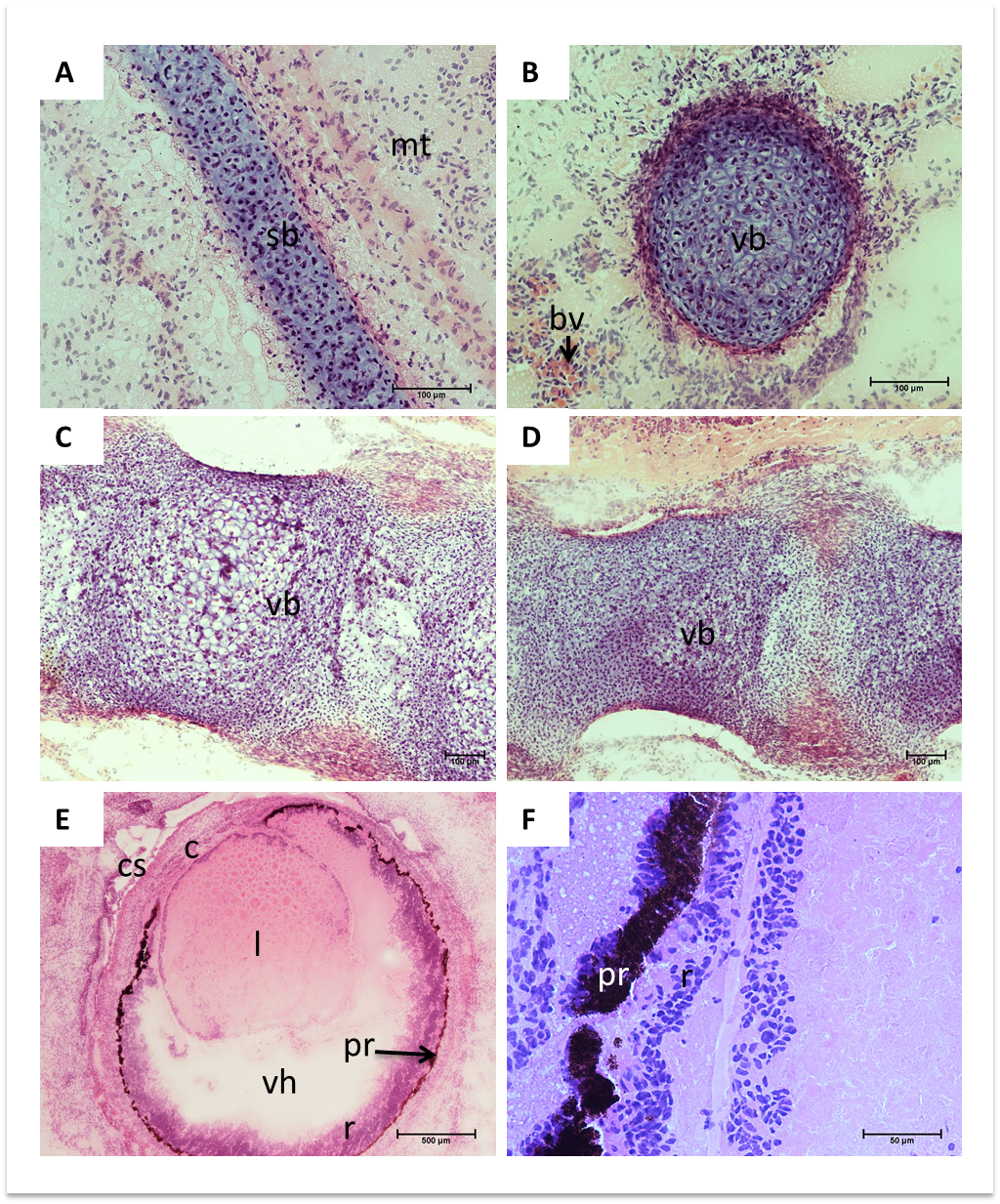
**

**Figure 2S:** Light microscopy of swine fetus at 50 days of gestation. **[A]** Ossification of skull bones (sb), which is surrounded by mesenchyme tissue (mt). **[B-D]** Identification of chondroblasts rounded in shape with a centrally nuclei during the ossification of the vertebral bodies (vb). The mesenchyme was richly vascularized by blood vessels (bv). **[E and F]** Eye; note vitreous humor (vh), lens (l), conjunctival sac (cs), cornea (c), retina (r), and pigmented layer of the retina (pr).


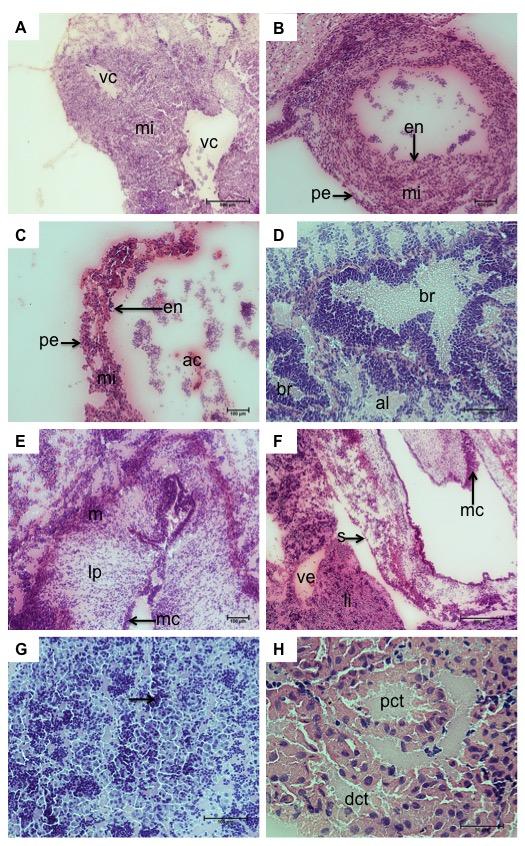


**Figure 3S:** Light microscopy of swine fetus at 50 days of gestation. **[A and B]** Ventricular chamber (vc) and **[C]** Atrial chamber (ac). Note the endocardium (en), myocardium (mi), and pericardium (pe). **[D]** Numerous bronchioles (br) and alveolar structures (al) were presented in the lung parenchyma. **[E and F]** The developing stomach showed the typical layers of the gastrointestinal tract: mucosa (mc), lamina propria (lp), muscularis (m), and serosa (s). In addition, note the relation of the stomach and liver (li). **[G]** Hepatoblasts (arrow) dispersed in the liver parenchyma. **[H]** Kidney showing proximal (pct) and distal convoluted tubules (dct).


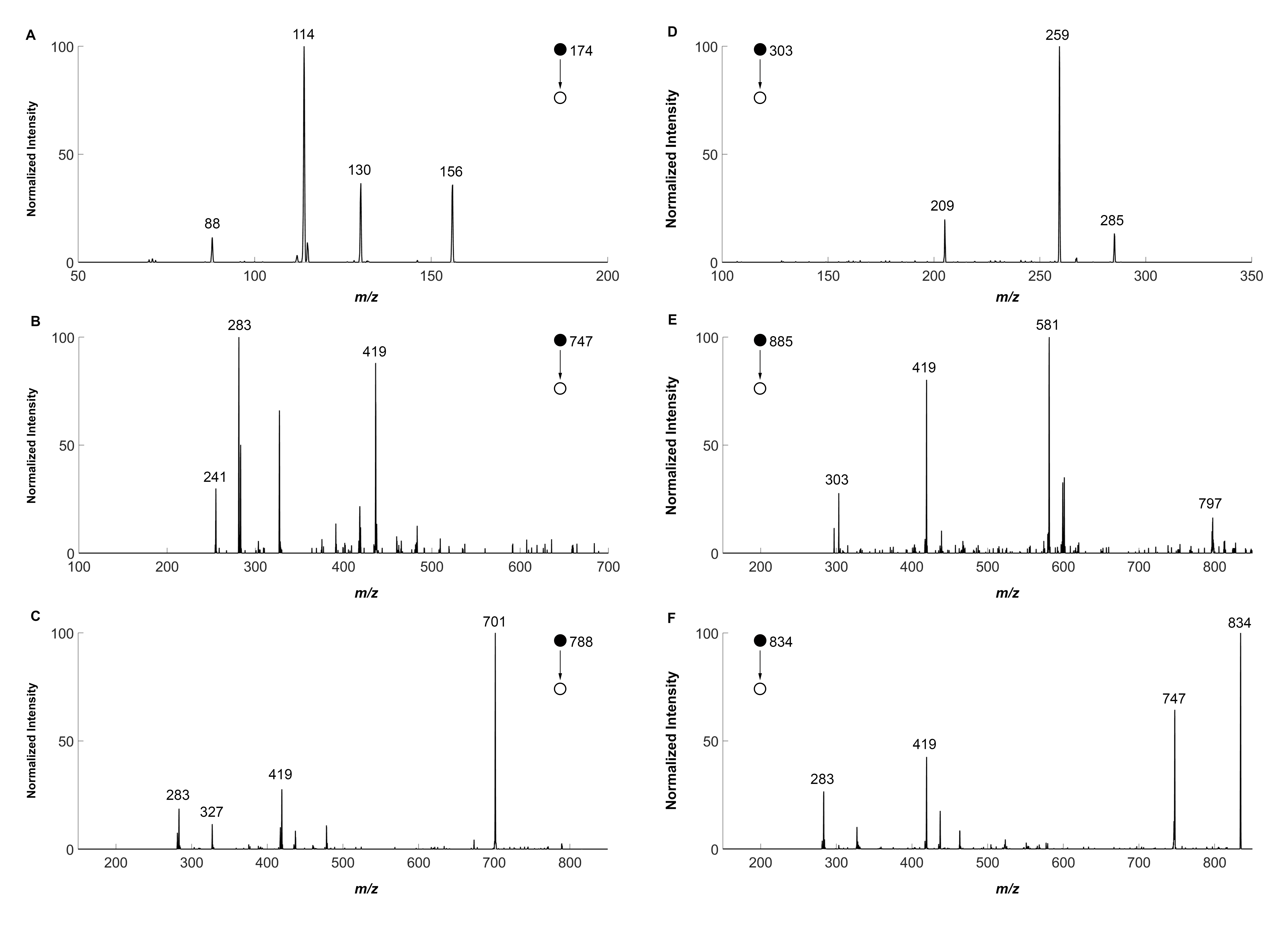


**Figure S4:** Product ion (MS/MS) scans acquired using DESI-MS in negative ion mode for precursor ions **[A]** *m/z* 174, N-acetyl aspartate; **[B]** *m/z* 303, arachidonic acid; **[C]** *m/z* 747, PG(34:1); **[D]** *m/z* 885, PI(38:4); **[E]** *m/z* 788, PS(36:1); **[F]** *m/z* 834, PS(40:6).
